# Automatic registration of 2D MR cine images for swallowing motion estimation

**DOI:** 10.1371/journal.pone.0228652

**Published:** 2020-02-11

**Authors:** Jinzhong Yang, Abdallah S. R. Mohamed, Houda Bahig, Yao Ding, Jihong Wang, Sweet Ping Ng, Stephen Lai, Austin Miller, Kate A. Hutcheson, Clifton Dave Fuller, for the Joint Head and Neck Radiotherapy MRI Development Cooperative

**Affiliations:** 1 Department of Radiation Physics, The University of Texas MD Anderson Cancer Center, Houston, TX, United States of America; 2 Department of Radiation Oncology, The University of Texas MD Anderson Cancer Center, Houston, TX, United States of America; 3 Department of Head and Neck Surgery, The University of Texas MD Anderson Cancer Center, Houston, TX, United States of America; University of Nebraska Medical Center, UNITED STATES

## Abstract

**Purpose:**

To automate the estimation of swallowing motion from 2D MR cine images using deformable registration for future applications of personalized margin reduction in head and neck radiotherapy and outcome assessment of radiation-associated dysphagia.

**Methods:**

Twenty-one patients with serial 2D FSPGR-MR cine scans of the head and neck conducted through the course of definitive radiotherapy for oropharyngeal cancer. Included patients had at least one cine scan before, during, or after radiotherapy, with a total of 52 cine scans. Contours of 7 swallowing related regions-of-interest (ROIs), including pharyngeal constrictor, epiglottis, base of tongue, geniohyoid, hyoid, soft palate, and larynx, were manually delineated from consecutive frames of the cine scan covering at least one swallowing cycle. We applied a modified thin-plate-spline robust-point-matching algorithm to register the point sets of each ROI automatically over frames. The deformation vector fields from the registration were then used to estimate the motion during swallowing for each ROI. Registration errors were estimated by comparing the deformed contours with the manual contours.

**Results:**

On average 22 frames of each cine scan were contoured. The registration for one cine scan (7 ROIs over 22 frames) on average took roughly 22 minutes. A number of 8018 registrations were successfully batch processed without human interaction after the contours were drawn. The average registration error for all ROIs and all patients was 0.36 mm (range: 0.06 mm– 2.06 mm). Larynx had the average largest motion in superior direction of all structures under consideration (range: 0.0 mm– 58.7 mm). Geniohyoid had the smallest overall motion of all ROIs under consideration and the superior-inferior motion was larger than the anterior-posterior motion for all ROIs.

**Conclusion:**

We developed and validated a deformable registration framework to automate the estimation of swallowing motion from 2D MR cine scans.

## Introduction

There is a growing population of head and neck cancer (HNC) survivors treated with curative doses of radiotherapy that may result in chronic radiation-associated-dysphagia (RAD) and aspiration [1, 2]. Much of this rise in incidence is represented by patients with human papilloma virus (HPV) associated disease, a subgroup of HNC typically diagnosed at a younger age with favorable prognosis for long-term survival [3]. This rapidly growing group of survivors has potential to live many years with the effects of radiotherapy. Thus, these survivors also face a substantive risk of lifelong swallowing dysfunction [4], a primary functional concern of this population [5], potentially resulting in late non-cancer mortality.

Magnetic resonance (MR) imaging is an effective tool to study aerodigestive tract motion and swallowing [6–9]. In the HNC radiotherapy population, 2D MR cine sequences of swallowing can evaluate motion in multiple regions-of-interest (ROI) with implications on both radiation therapy (RT) target margins in treatment planning and possibly the functionality of swallowing muscles longitudinally during and after radiotherapy. Existing approaches include pixel based and region-of-interest (ROI) based methods to estimate motion in manual fashion [8, 10]. There is, however, not an easy and robust approach to automate the entire process of motion estimation across multiple ROIs, representing a critical limitation to implementation of this imaging method in the clinical setting or across a large volume of patients in research studies.

In this study, we developed a comprehensive framework to automate the quantitative motion analysis for multiple swallowing ROIs using non-rigid registration approach. Multiple ROIs related to swallowing function were contoured from 2D MR image frames. A fully automated non-rigid registration, thin-plate spline robust point matching (TPS-RPM) algorithm, was used to register these ROIs from one frame to the other, to facilitate the quantitative motion analysis of these ROIs. This work represents the first attempt to automate the 2D MR cine image registration for swallowing motion estimation toward multi-ROI RT plan optimization and possibly RAD risk stratification.

## Materials and methods

### Patient data

**Fig 1:**
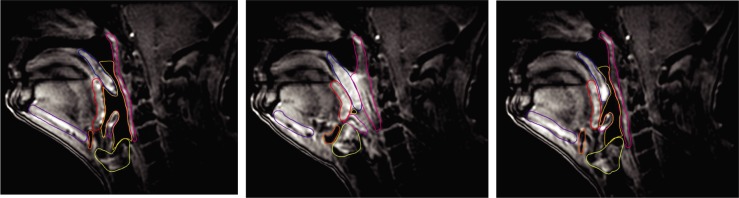
Illustration of swallowing related regions-of-interest (ROIs) contoured from the 2D MR cine scans. Contours at 3 typical states of swallow are shown. These ROIs were contoured on each frame from pre-swallow rest till offset. The contours shown include 7 swallowing related ROIs (pharyngeal constrictor [pink], epiglottis [magenta], base of tongue [red], geniohyoid [purple], hyoid [orange], soft palate [blue], and larynx [yellow green]) and air column (This figure is best viewed in electronic version).

This MRI analysis study was approved by the institutional review board of MD Anderson Cancer Center (protocol RCR03-0800) with a waiver of informed consent. Only MR cine scans contain the patient information in this study. The scans were acquired between October 2015 and January 2018 in our institution. These scan data were fully anonymized when the analysis was performed. We retrospectively identified 21 patients with 2D fast spoiled gradient-echo (FSPGR)-MR cine scans of the head and neck conducted through the course of definitive radiotherapy for oropharyngeal cancer. Included patients had at least one cine scan before, during, or after radiotherapy. A total of 52 cine scans were acquired, with 20 before, 15 during, and 17 after radiotherapy, respectively. Contours of 7 swallowing related regions-of-interest (ROIs), including pharyngeal constrictor (PC), epiglottis, base of tongue (BOT), geniohyoid muscle, hyoid bone, soft palate, and larynx, were manually delineated by one Radiation Oncologist (ASRM, HB, SPN) from consecutive frames of the cine scan covering at least one swallowing cycle (Fig 1). Temporal events in the swallow were tagged as pre-swallow rest, peak swallow, and offset states based on the volume of air column during a swallowing cycle. These are saliva swallows without a bolus.

### Acquisition parameters cine MRI

The MRI was performed on a MAGNETOM Aera 1.5T MR scanner (Siemens Healthcare, Erlangen, Germany) with two large four-channel flex phased-array coils. The cine MR scans were acquired in sagittal view with a temporal resolution 160 ms. Acquisition parameters for the cine MRI were: a true FISP imaging sequence, FOV = 25.6 cm, TR = 160 ms, TE = 1 ms, pixel size = 1 × 1 mm^2^ in-plane, flip angle = 60°, bandwidth = 1500 Hz/pixel, slice thickness = 15 mm, single slice, and 128 × 128 matrix with parallel imaging factor 2. The average acquisition time was 80 seconds.

### Automated contour registration

We applied a modified thin-plate-spline robust-point-matching (TPS-RPM) algorithm[11] to register the point sets of each ROI automatically over frames. The TPS-RPM algorithm[12] was originally developed to solve the point matching problem in the presence of outliers, which are points in one point set which have no correspondence in the other point set. The TPS-RPM algorithm is able to identify these outliers and establish correspondence for the corresponding points only. To achieve this goal, TPS-RPM algorithnm takes advantage of the softassign technique and the deterministic annealing technique. The softassign technique[13] allows fuzzy correspondence at the beginning of the matching process, and gradually enforces the correspondene to be binary when the algorithm converges. Deterministic annealing technique[14] allows only a relatively rigid transform at the beginning of the matching process, and gradually inceases the nonrigidity of the transform at a later stage. This helps to determine an optimal binary correspondence and overcome local optimum during the matching process, in particular, when large deformations are present. The modified TPS-RPM algorithm[11] can robustly handle outliers present in both point sets, making the registration of ROI over frames very reliable. In addition, TPS-RPM algorithm results in analytical solutions, which makes the optimization for point matching very fast. In our implementation, points of each ROI were resampled to a uniformed grid based on the image resolution and rescaled to a unit box. The modified TPS-RPM algorithm was then used to register the points of each ROI between every two consecutive frames. The resulting transformation was then rescaled back to the original scale and applied to the uniform grid, to generate a deformation vector at each grid point. Deformation vectors at those grid points inside the specific ROI were used to calculated the motion for that ROI. For motion between any two frames, we concatenated the deformation vectors for all consecutive frames that connect those two frames to generate deformation vectors between these two frames. The contour registration was implemented in Matlab 2017b (Mathworks, Natick, MA).

### Motion estimation

The aformentationed deformation vectors from the registration were then used to estimate the motion during swallowing for each ROI. Similar to a previous study,[15] for each grid point inside each ROI, the deformation vector was orthogonally decomposed into anterior-posterior (AP) and superior-inferior (SI) directions to estimate the motion in anterior, posterior, superior, or inferior direction. The motion magnitude in each direction for one ROI was calculated as the average motion of all grid points inside the ROI. The ROI motion at each frame was calculated as the displacement compared with the initial frame that was contoured. For a specific patient, the maximum value of the motion magnitudes of all contoured frames at each direction for a specified ROI was used to characterize the motion behavior of the ROI.

### Automatic validation

The contour registration and motion estimation are fully automated, without the need of any human interaction. We evaluated the time needed for each registration and the registration error by comparing the registered (deformed) contour with the manual contour for validation. The registration error of one ROI was defined as the average of all distance between the registered point and the reference manual point on the ROI. Most registration should show a small error (usually ≤ 1 mm). By analyzing the registration errors of all contour registrations, we were able to automatically detect the unsuccessful registrations. For these unsuccessful registrations, manually tweaking parameters of the registration algorithm was needed and usually was able to achieve a registration error within 2 mm. In our experiments, the unsuccessful registration was rare; and in our analysis, we only analyzed the original automatic registration results.

## Results

### Automatic contour registration

**Fig 2:**
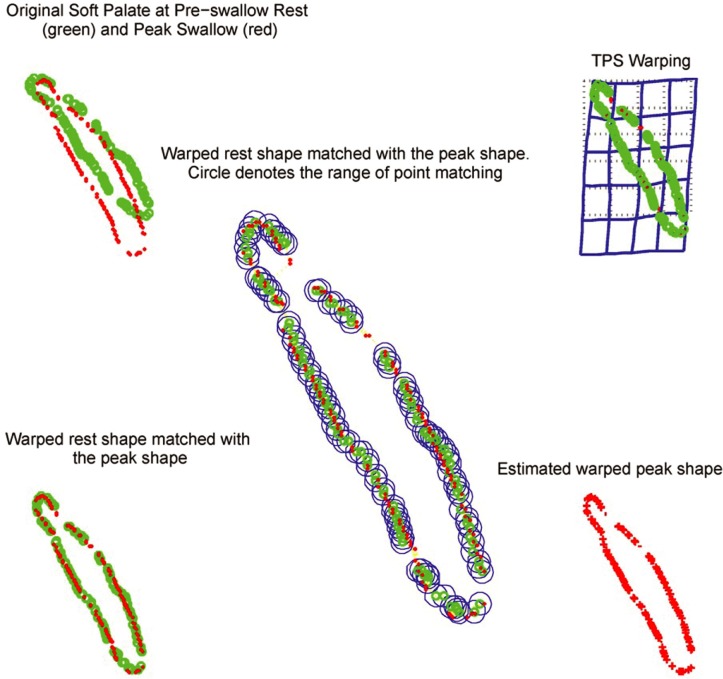
Registration of the soft palate from pre-swallow state to the peak swallow state using the modified thin plate spline robust point matching (TPS-RPM) algorithm.

On average 22 frames (range: [8, 57]) of each cine scan were contoured for a swallowing cycle. The average time for registration of one cine scan (7 ROIs over 22 frames) was approximately 22 minutes. A total of 8018 contour registrations were batch processed without human interaction after the contours were drawn. Of the 8018 registrations, one PC contour registration resulted in an error >1000 mm, indicating a complete fail in the registration. A close check showed that the PC registration was failed for that patient due to the narrow elongate shape of PC contours, which resulted to reflex in registration (the left side of one contour was registered to the right side of another contour and vice versa). Fine tuning of registration parameters was needed to overcome this issue. Of the other registrations, one registration (BOT contour) had an error of 2.06 mm, 62 registrations (all PC contours) had an error between 1 mm and 2 mm, and all other registrations (99.2%) had an error less than 1 mm. The registration of PC contour was in general less accurate than other contours, possibly due to its narrow elongate shape. By excluding the single failed registration (out of 8018), the average registration error for all ROIs and all patients was 0.36 mm (range: 0.06 mm– 2.06 mm). Fig 2 illustrates the registration of the soft palate using the modified TPS-RPM algorithm.

### Motion estimation

**Fig 3:**
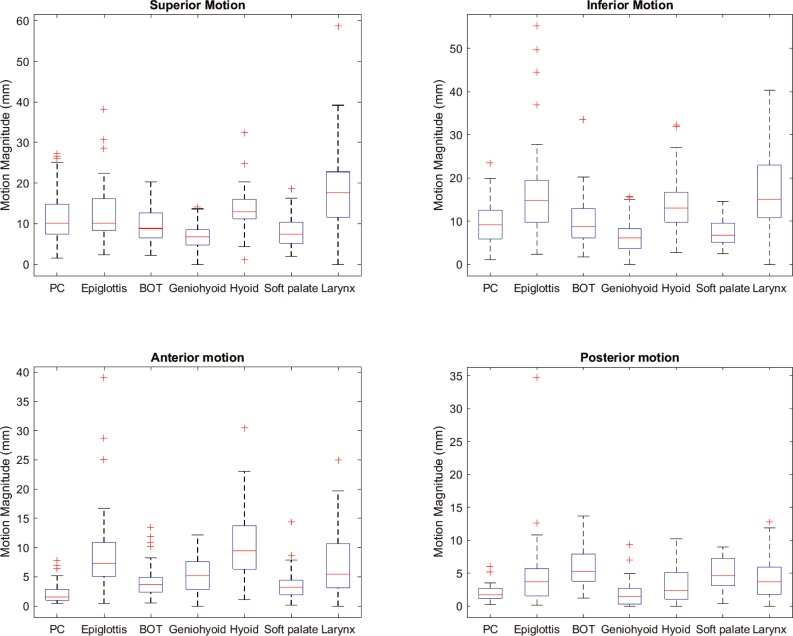
Boxplot of the swallowing structure motions in the four directions (superior, inferior, anteriror, and posterior) for 52 cine scans of 21 patients. Abbreviations: PC–pharyngeal constrictor; BOT–base of tongue.

**Fig 4:**
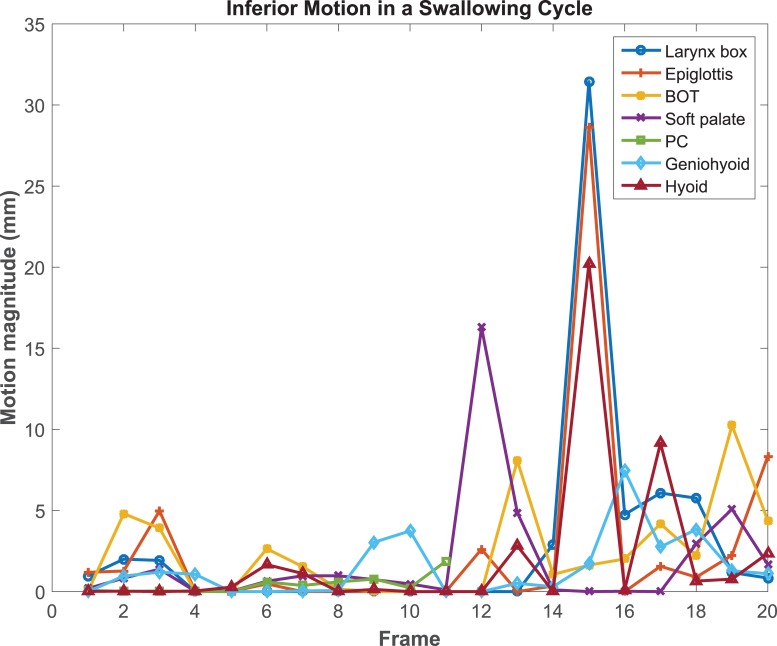
The inferior motion of 7 swallowing related ROIs estimated from the contour registration over 20 fames for one patient. It shows that the peak swallow happened at the 15^th^ frame in this MR cine scan. The larynx and epiglottis have the largest motion magnitude of about 30 mm.

The average motions of the 7 ROIs in each four directions of the 21 patients are listed in Table 1. The motion data were averaged over all frames from 52 cine scans, including before, during, and after radiotherapy scans. The boxplot of the motion data are shown in Fig 3. In analyzing the PC motion, we excluded the failed PC registration scans for that patient. For all ROIs, the superior-inferior direction exhibited larger motion than the anterior-posterior direction. Geniohyoid overall showed the smallest motion while larynx showed the largest motion among all 7 ROIs. The largest motion on average is from larynx in superior direction of 18.4 mm (range: 0 mm– 58.7 mm). For each cine scan, ROI motions in each direction can be plotted over time as a function of contoured frames, as shown in Fig 4. From this figure, one would be able to identify the time of peak to rest motion event and identify the ROIs that were potentially good for motion analysis.

**Table 1:** Average motion of the 7 regions of interest in the four directions (superior, inferior, anteriror, and posterior) over all frames.

	Mean motion ± standard deviation (min, max); mm^a^						
**Motion direction**	pharyngeal constrictor	epiglottis	base of tongue	geniohyoid	hyoid	soft palate	larynx
**Superior**	11.1 ± 6.3 (1.5, 27.3)	12.9 ± 6.8 (2.4, 38.2)	9.8 ± 4.2 (2.2, 20.3)	6.7 ± 3.2 (0.0, 14.2)	13.6 ± 5.3 (1.1, 32.4)	8.3 ± 4.2 (1.9, 18.7)	18.4 ± 10.0 (0.0, 58.7)
**Inferior**	9.6 ±5.0 (1.0, 23.5)	16.4 ± 10.8 (2.3, 55.1)	9.8 ± 5.5 (1.7, 33.5)	6.3 ± 3.4 (0.0, 15.7)	13.6 ± 6.2 (2.7, 32.3)	7.3 ±3.1 (2.5, 14.5)	17.3 ± 9.2 (0.0, 40.3)
**Anterior**	2.2 ± 1.7 (0.4, 7.8)	8.8 ± 6.9 (0.4, 39.0)	4.2 ± 2.7 (0.5, 13.5)	5.3 ± 3.2 (0.0, 12.1)	10.6 ± 6.5 (1.0, 30.5)	3.6 ± 2.4 (0.1, 14.4)	7.0 ± 5.5 (0.0, 25.0)
**Posterior**	2.0 ± 1.1 (0.2, 6.0)	4.6 ± 5.1 (0.2, 34.7)	6.0 ± 2.8 (1.2, 13.7)	1.8 ± 1.9 (0.0, 9.3)	3.0 ± 2.7 (0.0, 10.2)	4.9 ± 2.4 (0.4, 9.0)	4.2 ± 3.3 (0.0, 12.8)

^**a**^The motion values were calculated from 52 cine scans of 21 patients shown as mean motion ± standard deviation (min, max) with the unit of mm.

## Discussion

This report presents an automated approach to register contours and estimate motion from swallow MR cine images. This tool facilitated the batch analysis of motion during swallowing from cine MR images acquired longitudinally before, during, and after radiotherapy for HNC. As aforementioned, existing approaches include pixel based and ROI-based methods to estimate motion are mainly in manual fashion [8, 10]. By automating the motion-quantification process, we greatly improve the efficiency of swallowing motion analysis, and potentially reduce the intra- or inter-observer variability in quantifying the motion. In addition to the ROI-based registration approach, other approach may also be employed for the motion analysis, for example, the image-based deformable registration approach [16, 17] or the optical-flow based tracking approach [18].

The implications of this work include potential for dysphagia risk reduction or risk stratification using MR imaging. That is, if it becomes technically efficient to quantify swallowing motion from cine MR images, it may be possible to personalize RT margins for individual’s swallowing motion or identify subclinical changes in swallowing muscle motion before clinically detectable dysphagia manifests. Since cine sequences are easily added to standard surveillance and RT planning MR image acquisitions, a tool to register and quantify motion efficiently opens possibilities for clinical translation that are not practical when time consuming manual post-processing is required.

One potential application of this tool might be personalized margin reduction in RT planning. The last decade saw great strides in efforts to prevent RAD. Dysphagia-optimized planning constraining dose to pharyngeal constrictors and the larynx decreases risk by 6% [19]. Further optimization without advanced image guidance is likely to be limited by the inherent overlap of swallowing avoidance parameters and RT target volumes given the close proximity of tumor and swallowing critical anatomy in the head and neck region. Samuels et al [20] demonstrate that moving from a standard planning target volume (PTV) to a reduced target volume has greater impact on normal tissue dose than a 10-Gy reduction in PTV prescription. Thus, fundamentally, dose de-escalation is insufficient to reduce normal tissue dose relative to margin reduction. We contend that margin reduction is a key strategy to lower non-target dose better than OAR dose constraints. Yet, current margin reduction strategies are handicapped by assumption of uniform motion across heterogenous non-target structures. This technical method, therefore, facilitates testing of using variable multi-ROI swallowing motion parameters to safely reduce margins in RT planning. As noted, the swallowing motion likely happens randomly during the radiation delivery. The motion estimated from the cine images could be treated as random uncertainties to generate personalized margins using the existing margin recipe [21–23]. With FDA clearance of MR-LinAc, adaptive MR imaging based planning methods have real potential for clinical application. The authors propose *in silico* testing of this RT planning technique as a next step.

In addition to RT planning applications, quantification of swallowing motion on cine MR imaging (serially through treatment and survivorship) might provide a surrogate toxicity endpoint of dysphagia or a method to risk stratify for dysphagia early (e.g., during RT) when subclinical change is likely happening prior to clinically detectable pharyngeal dysfunction. Deep learning techniques, for instance, have shown ability to discriminate healthy volunteers from postsurgical (partial glossectomy) HNC survivors based on MR imaging tongue motion parameters during speaking tasks [6]. Our tool facilitates testing of similar discriminant capacity of motion from functional swallowing units or swallowing muscle ROI over the cancer treatment trajectory of individual survivors. In a parallel study, we are investigating the change in motion over the time (before, during, and after radiotherapy) as a potential predictor of dysphagia for head and neck cancer survivors.

While this technical report represents major step toward automated motion estimation, the remaining major limitation is the need to manually contour the ROI for motion analysis. Manual segmentation is time consuming. Thus, a future step includes development of an auto-contouring tool or use of our existing data to train a model for auto-contouring to combine registration and segmentation for auto-processing the MR sequences for motion estimation during swallowing. Another limitation is that motion estimation is from all frames over the course of swallowing and not linked to a particular physiologic event of interest (e.g., peak pharyngeal constriction or post-swallow rest). Temporally aligning the motion data represents an opportunity for future work. Further, the 2D cine images used in this study is in the sagittal plane, and only the SI and AP motion was studied. However, for asymmetric irradiation (e.g. unilateral irradiation or bilateral irradiation with differential dosing on each side), the left-right (LR) motion might be of interest for swallowing motion study. In this situation, it is necessary to analyze coronal or axial cine MR images. The framework developed in this study can be applied to these images as well.

## Conclusions

We developed and validated a deformable registration framework to automate the estimation of swallowing motion from 2D MR cine scans. This work laid the foundation for future multi-ROI RT plan optimization and possibly RAD risk stratification.

## Supporting information

S1 File The motion value details for the 7 regions of interest (pharyngeal constrictor, epiglottis, base of tongue, geniohyoid, hyoid, soft palate, and larynx) in 52 cine scans of 21 patients. (XLSX)
